# Editorial: The Roles of Oncogenic Phosphatase/Kinase in Tumors

**DOI:** 10.3389/fcell.2022.878868

**Published:** 2022-03-09

**Authors:** Mirco Galiè

**Affiliations:** Department of Neuroscience, Biomedicine and Movement, University of Verona, Verona, Italy

**Keywords:** cancer, tumor, protein kinase, protein phosphatase, signaling

Protein kinases and phosphatases are antagonistic mediators of signal transduction pathways, which function through the phosphorilation/dephosphorylation, respectively, of their specific downstream targets. The proper balancing of protein kinases/phosphatases activity is crucial to maintain cell homeostasis and assure the correct timing, amplitude and duration of environmental signals underlying biological process such as cell growth, differentiation, migration, metabolism, survival and death.

Dysregulation of the complex network of protein kinase/phosphatase activity has long been recognized as an intrinsic hallmark of tumor transformation and progression, either as primary cause or consequence. Decades of research on this topic have allowed to identify hundreds of kinases and phosphatases in human genome (Hooft van Huijsduijnen, 1998) and the abnormal activity of many of them have been shown to underly tumorigenesis in multiple types of cancer (Stebbing et al., 2014).

In this research topic, experts in the field contributed with either original research or review articles to move forward our knowledge on interesting aspects of the complex role of kinases and phosphatases in tumorigenesis.

Dong et al. summarized how the gain-of-function mutation of Src homology region 2 protein tyrosine phosphatase 2 (SHP2) might contribute to promote cancer progression both through cell-autonomous and non-cell-autonomous mechanisms. They also discussed the role of SHP2 mutations in drug resistance and the potential therapeutic use of small molecule SHP2 inhibitors in anti-cancer therapies.

Huang et al. showed how the cyclin-dependent kinase 9 (CDK9) inhibitors might induce the apoptosis of B-cell acute lymphocytic leukemia (B-ALL) by inhibiting c-Myc-mediated glycolitic metabolism.

Ciummo et al. showed that the chemokine C-X-C motif ligand-1 (CXCL-1) functions as an autocrine growth factor which promotes immune escape and sustains breast cancer stem cell phenotype and epithelial-to-mesenchymal transition, two intrinsically interrelated aspects of the most aggressive breast cancers.

Turdo et al. reviewed the major protein kinase and phosphatase pathways which impact on the capability of CSCs to evade normal physiological constraints on survival, growth, and invasion. They also discussed the potential use of phosphatase/kinase inhibitors in counteracting CSCs expansion during cancer development and progression.

Centoze et al. reviewed 15 years of research about the role of the protein p130Cas to function as adaptor multiprotein signaling complexes which sustain breast cancer progression through pleiotropic effects on cell motility, cell adhesion, cytoskeleton remodeling, invasion, survival, and proliferation. They also discussed the p130Cas-antagonistic role of p140Cap, which associate with p130Cas through interaction with the Src kinase and display well established anti-tumor effects in breast cancer and neuroblastoma.

Hao et al. reviewed the controversial role in leukemia of two protein tyrosine phosphatases, SH2 domain-containing phosphatases 1 and 2 (SHP-1 and SHP-2) and of the phosphatase inhibitor SH2-domain-containing inositol phosphatase.

Yao et al. provided the transcriptional profiling of tumor-associated protein kinases and phosphatases and other phosphorylation-related genes in samples of human hepatocellular carcinoma, thus identifying the overexpression of a set of protein kinases and phosphorylation-related genes that were associated to cancer stem cell phenotype and poor clinical outcome.

Li et al. identified 6-Phosphogluconolactonase overexpression as a marker of poor prognosis in hepatocellular carcinoma (HCC) and showed that the downregulation of this gene was able to impair cell proliferation, migration and invasion capability of HCC likely inhibiting ROS-mediated apoptosis.

Boni and Sorio discussed the role of protein tyrosine phosphatase gamma (PTPRG) as natural counterpart of tyrosine kinases, and reviewed how its loss-of-function has been reported in many types of cancers, such as Lymphoma and Leukemia, colorectal, nasopharyngeal, ovarian, breast, lung, gastric cancer.

The understanding of the effects of kinases/phosphatases in cancer and their molecular mechanisms of action has been greatly increased over the last decades and hold promise in therapy (Vainonen et al., 2021). Nevertheless, a deeper understanding of this complex network of cancer regulators is still needed to design more effective and specifically targeted strategies of treatment which might contribute to eradicate the infinite variants of this pandemic disease that disseminates death all over the world.
